# The Seroprevalence of Severe Fever with Thrombocytopenia Syndrome: An Epidemiological Study of Korean Veterinary Hospital Workers

**DOI:** 10.3390/v15030609

**Published:** 2023-02-23

**Authors:** Choon-Mee Kim, Dong-Min Kim, Mi-Seon Bang, Jun-Won Seo, Na-Ra Yun, Da-Young Kim, Mi-Ah Han, Ji-Hye Hwang, Sook-Kyung Park

**Affiliations:** 1 Premedical Science, College of Medicine, Chosun University, Gwangju 61452, Republic of Korea; 2 Department of Internal Medicine, College of Medicine, Chosun University, Gwangju 61452, Republic of Korea; 3 Department of Preventive Medicine, College of Medicine, Chosun University, Gwangju 61452, Republic of Korea; 4 Division of Control for Zoonotic and Vector Borne Disease, Korea Disease Control and Prevention Agency, Cheongju-si 28159, Republic of Korea

**Keywords:** epidemiology, seroprevalence, severe fever with thrombocytopenia syndrome virus, severe fever with thrombocytopenia syndrome, veterinary hospital

## Abstract

Severe fever with thrombocytopenia syndrome (SFTS) is a zoonotic tick-borne infectious disease caused by the SFTS virus (SFTSV). Few studies have assessed SFTS seroprevalence among veterinary hospital staff and their awareness of SFTS. From January to May 2021, serum samples from 103 veterinary hospital staff were tested for SFTS using an enzyme-linked immunosorbent assay (ELISA), an immunofluorescence assay, and a 50% plaque reduction neutralization antibody test, which yielded positive results in four (3.9%), three (2.9%), and two (1.9%) participants, respectively. A questionnaire was used for an epidemiological investigation. ELISA positivity was higher among those who lacked awareness of possible animal-to-human SFTS transmission (*p* = 0.029). SFTS awareness was significantly lower among veterinary hospital staff than among the veterinarians (*p* < 0.001). Providing staff with training concerning standard precautions and the use of appropriate personal protective equipment is important.

## 1. Introduction

Severe fever with thrombocytopenia syndrome (SFTS) is a zoonotic tick-borne infectious disease caused by the SFTS virus (SFTSV) (genus *Banyangvirus*; family Phenuiviridae) and is mainly seen in Korea, China, and Japan [1], with the number of human cases increasing annually in these countries. The reported case fatality rate associated with SFTSV infections varies greatly, ranging from 5.3% to 16.2% in China to 27% in Japan and 23.3% in South Korea [2,3]. Antibodies to SFTSV have been detected in wild and domestic animals such as goats, deer, cattle, dogs, and cats in the SFTS endemic areas of these three countries. In Japan, SFTSV transmission through direct contact with bodily fluids of sick companion animals such as cats and dogs has been reported [3]. The SFTS seroprevalence in Korea is known to range from 2.1% to 4.1%, based on indirect immunofluorescence assay (IFA) and enzyme linked immunosorbent assay (ELISA) tests. In China, the SFTS seroprevalence has been reported to range from 3.3% to 9.2% based on ELISA analyses [4].

Recently, genetic and phylogenetic analyses have identified at least six genotypes (A-F) of SFTSV in certain East Asian countries, and the prevalence of each SFTSV genotype varies across these countries. In China, genotype F (44.3%) is the most prevalent genotype, followed by genotypes A and B. In Japan and Korea, genotype B (especially genotype B-2 among the subtypes B-1, B-2, and B-3) is the most common [2].

SFTSV is transmitted by ticks. Human-to-human transmission due to close contact with blood and bodily secretions, and nosocomial transmission due to needle-stick injuries, has been reported [5,6]. Additionally, SFTSV can be transmitted by companion animals such as dogs and cats that have tested positive for SFTSV genes, with antibodies detected in such animals. Among veterinarians and animal caretakers, cases of secondary infection from companion animals with SFTS have been reported such as the case of two veterinarians who acquired SFTSV infection from treating cats, with whole-genome sequences of the SFTSV isolates being 100% identical [7,8,9]. Hence, SFTSV may be transmitted directly from animals to humans, and is not limited to tick-borne transmission [10].

Therefore, this study aimed to investigate SFTS seroprevalence among veterinarians and veterinary hospital staff treating companion animals to determine whether the staff had been exposed to SFTSV. To date, few studies have assessed SFTSV exposure in humans working with animals. We assessed the anti-SFTSV seroprevalence and analyzed the SFTS awareness and preventative behaviors in veterinary hospital workers, an occupational group potentially at high risk of SFTS.

## 2. Methods

From January to May 2021, blood samples were collected from veterinarians, animal caretakers (veterinary technicians), and administrative staff at local veterinary hospitals who had applied to participate in the SFTS Pilot Project of the Korea Disease Control and Prevention Agency. Those who voluntarily agreed to participate were included, and a questionnaire and blood collection were administered.

The study protocol was approved by the Institutional Review Board of Chosun University (permit no. 2020-11-022). All participants provided written informed consent to participate in the study. All experiments were performed in accordance with the relevant guidelines and regulations.

Based on a literature review of relevant studies published in Korea and other countries, a questionnaire was developed covering possible risk factors for SFTS infection, preventative behaviors, general characteristics, the occupational and living environment, prevention activity-related characteristics, and SFTS awareness. Venous blood samples were collected from the participants. The collected blood was transported to the laboratory at Chosun University Hospital. IFA and ELISA tests, and a 50% plaque reduction neutralization antibody test (PRNT_50_) were performed on the serum, according to the methods used in previous studies [9,10,11].

To detect immunoglobulin G (IgG) and IgM using IFA, twofold serial dilutions of the serum samples were reacted with SFTS antigen slides as primary antibodies, followed by fluorescein isothiocyanate-conjugated anti-human IgG and IgM (MP Biomedicals, Illkirch, France) as secondary antibodies. IFA IgG and IgM antibody titers ≥1:32 were used as positive cut-off values based on results from 15 health check-up participants at Chosun University Hospital.

The ELISA was based on the *E. coli* expressed nucleoprotein (NP) antigen of the SFTSV and a positive optical density (OD) cut-off value of 0.92, based on the OD_450_ + 3 standard deviations using seven health check-up participants at Chosun University Hospital. For the indirect ELISA, 96-well ELISA microplates were coated with 2 μg/mL of NP antigen protein, and reacted with 100-fold diluted serum samples as primary antibodies, followed by the HRP-conjugated goat anti-human IgG (H + L) secondary antibody (Cat# 628420,Invitrogen, Waltham, MA, USA). After reacting with 3,3′,5,5′-tetramethylbenzidine substrate (TMB; Sigma-Aldrich, Burlington, MA, USA), the reaction was stopped with 1 M H_2_SO_4_, and the absorbance was measured at 450 nm. The serum samples were tested in triplicate.

The PRNT_50_ was performed in a biosafety level 3 laboratory at the Health and Environment Research Institute of Gwangju City. The PRNT_50_ was carried out via inoculation of the serum sample with SFTSV and the infection of cells (Vero E6). The cells were then cultured, and plaque formation was examined. In brief, in a 24-well plate, Vero E6 cells were seeded at a density of 2.5 × 10^5^ cells/well in a cell culture medium (Dulbecco’s modified Eagle’s medium [DMEM], 10% of heat-inactivated fetal bovine serum [FBS], and 1% of a penicillin-streptomycin solution). The cells were kept in an incubator at 37 °C and 5% CO_2_ for one day. Serum was inactivated at 56 °C for 30 min, and fourfold serial dilutions with cell culture medium were performed. SFTSV and the diluted serum were mixed in a 1:1 (*v*/*v*) ratio and reacted at 4 °C for 1 h. Then, 100 µL of SFTSV and diluted serum were added into each well containing Vero E6 cells, and the cells were infected for 1 h. Next, 1 mL of the infection cell medium (DMEM, 5% of FBS, and 1% of methylcellulose) was added into each well, and the cells were incubated for 14 days at 37 °C in a 5% CO_2_ incubator. The infected cells were fixed and inactivated using a fixation reagent (acetone: methanol in a 1:1 ratio) and stained with 1% crystal violet for macroscopic confirmation of plaque formation. After calculation of the number of inhibited plaques at each serum dilution relative to the number of plaques in the well without the participants’ serum (but with the virus), nonlinear regression analysis was performed to determine the dilution factor that reduced the number of plaques by 50% as the final PRNT_50_ value. The neutralizing antibodies against SFTSV were then measured with the PRNT_50_ according to the methods used in a previous study [12,13]. Nonlinear regression analysis was performed using GraphPad Prism 8.0.1 (GraphPad Software, San Diego, CA, USA) [12,13]. To confirm acute SFTSV infection, viral RNA was isolated from the serum samples using the Viral Gene-Spin Viral DNA/RNA Extraction Kit (Intron; Seongnam, Korea). Conventional and nested polymerase chain reaction (PCR) or real-time (RT)-PCR testing were carried out as described previously [14].

General and work-related characteristics of the veterinary hospital workers were presented as frequencies and percentages. Fisher’s exact test was conducted to assess the association between the investigated putative risk factors and anti-SFTSV antibody positivity. SAS Version 9.2 (SAS Institute Inc., Cary, NC, USA) software was used for the statistical analysis.

Antibody positivity was defined as follows: IFA IgG and IgM titers of ≥1:32, ELISA IgG positivity (OD > 0.92), or PRNT_50_ positivity (titer > 1:10).

## 3. Results

**Figure 1:**
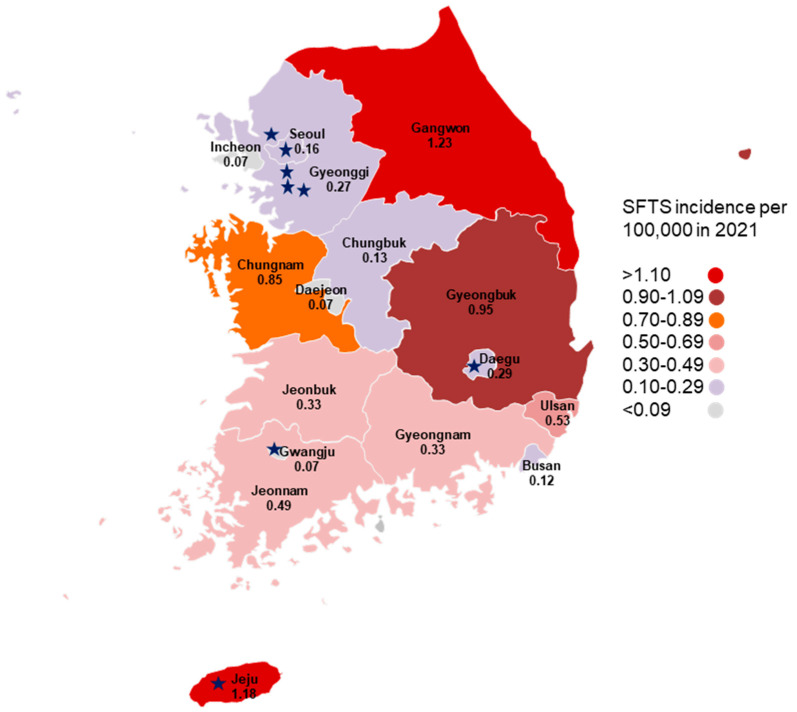
A choropleth map showing the incidence of SFTS per 100,000 population according to each region in 2021 and the participating veterinary hospitals’ locations (★). SFTS, severe fever with thrombocytopenia syndrome.

Blood samples were collected from 103 veterinary hospital workers in Seoul Special City, Gyeonggi Province (Seongnam City, Bundang City, Yongin City, Goyang City), Daegu Metropolitan City, Gwangju Metropolitan City, and Jeju Province (Figure 1). The SFTS incidence per 100,000 in 2021 is shown. In 2021, the SFTS incidence per 100,000 population in each study region was 1.18 in Jeju Province (eight cases), 0.29 in Daegu Metropolitan City (seven cases), 0.27 in Gyeonggi Province (37 cases), 0.16 in Seoul Special City (15 cases), 0.07 in Gwangju Metropolitan City (one case), and 0.33 nationwide (172 cases), respectively (https://www.kdca.go.kr/npt/biz/npp/ist/simple/simplePdStatsMain.do).

This study comprised 21 men (20.4%) and 82 women (79.6%), with women aged 20–29 years comprising the largest demographic group (46/103, 44.7%) (Table 1).

ELISA, IFA, and PRNT_50_ analyses yielded positive results in four (3.9%), three (2.9%), and two (1.9%) participants, respectively. The occupations in the study population included veterinarians (40.8%), animal caretakers (42.7%), administrative workers (8.7%), and others (7.8%). In three (2.9%) participants, the serum anti-SFTSV IFA IgG titer was ≥1:32, indicating a positive result. Of these three participants, two had serum anti-SFTSV IFA IgG titers ≥ 1:64. The IFA immunoglobulin M (IgM) antibody titer was <1:32 in the serum samples of all 103 participants (Table 2).

The SFTS ELISA test results yielded an OD value greater than the cut-off value of 0.92 in the serum of four (3.9%) participants. Two of the four participants with positive ELISA results had elevated IFA IgG titers (1:256 and 1:64). Moreover, two participants were seropositive in the IFA and ELISA analyses, and were also positive for neutralizing antibodies determined using PRNT_50_. The IFA antibody titer was ≥1:64. The neutralizing antibody titers of these two participants on PRNT_50_ were 1:52 and 1:136 (Supplementary Figure S1). A PCR test was performed to test for SFTSV genes. All the participants tested negative, confirming that none of the participants had acute SFTSV infection. SFTS awareness was assessed according to seropositivity on each of the three assays (Table 3).

Five (4.9%) participants were vaccinated against influenza in 2020, of whom one (20%) participant tested IgG positive in the ELISA analysis. SFTS awareness was assessed in relation to occupation. All of the veterinarians had heard of SFTS. In contrast, 45.5% of the animal caretakers had heard of SFTS but were not very familiar with the disease, and 29.6% had not heard of SFTS. Of the animal caretakers, 52.3% responded that SFTS could be transmitted from animals to humans, and 45.5% were unsure. Among the administrative workers, 44.4% considered that SFTS could be transmitted from animals to humans, and another 44.4% were unsure (Table 4).

Moreover, most participants did not wear gloves (89.3%) or goggles (99%) during animal care. Interestingly, those who tested positive for anti-SFTSV IFA IgG and ELISA IgG antibodies only included those who did not wear gloves and goggles during treatment and who did not change their protective clothing after individual treatment, as shown in Table 5.

Among the 103 participants, three (2.9%) tested positive for anti-SFTSV IgG on IFA. Of these, two tested positive for anti-SFTSV IgG in ELISA, and two tested positive for neutralizing antibodies on PRNT_50_. Among the 87 participants who reported exposure to animal blood, two tested positive for anti-SFTSV IgG in IFA, three tested positive for anti-SFTSV IgG in ELISA, and two tested positive for neutralizing antibodies in PRNT_50_.

Two participants suspected that they had become infected with SFTSV when treating a Samoyed dog in October 2019. At the time, one veterinarian at a veterinary hospital, who did not participate in this study, was infected with SFTSV. The results of an epidemiological investigation of a 29-year-old female animal caretaker who tested positive for SFTSV-neutralizing antibodies indicated that she had removed ticks from the Samoyed dog with her bare hands while providing an oxygen hose. The Samoyed dog had poor breathing and a tracheal tube was inserted and supported with mechanical ventilation. The dog vomited blood, and the tracheal tube was expelled by the dog when spontaneous breathing occurred (Supplementary Figure S2). At the time, the animal caretaker was wearing a mask without goggles and placed her ungloved hands in the dog’s mouth and wiped down the vomitus with gauze. The animal caregiver subsequently developed myalgia, headaches, dizziness, and digestive problems and visited a local emergency room with a fever of 39 °C, diarrhea, a swollen throat, and pain, on 13 October 2019. A 26-year-old female animal caretaker who tested positive for SFTSV-neutralizing antibodies, but who did not report any SFTS-related symptoms, also removed vomited blood from the dog’s mouth with her bare hands.

## 4. Discussions

In 2016, a woman in her fifties from Yamaguchi Prefecture in Japan developed symptoms of fever, loss of appetite, vomiting, and general fatigue two days after being bitten by a sick cat. She died of multi-organ failure, and an autopsy was conducted to determine the cause of death. A lymph node biopsy at the site of the cat bite confirmed necrotizing lymphadenitis with hemophagocytosis, and the immunohistochemistry results indicated the presence of SFTSV in the lymph nodes [15]. In addition to cat-to-human transmission, dog-to-human transmission of SFTSV has also been reported. In 2017, SFTSV infection was confirmed virologically in a four-year-old dog with lethargy and anorexia in western Japan. The dog’s owner, a man in his forties, cared for a sick dog and became sick 10 days after SFTS onset in the dog, presenting with symptoms such as fever, joint pain, headache, nausea and vomiting as well as leukopenia and thrombocytopenia. He was diagnosed with SFTS through IgM detection and neutralizing antibodies against SFTSV, indicating that humans may be at risk of SFTSV infection through direct contact with sick dogs infected with SFTSV [16].

In Miyazaki Prefecture, Japan, an epidemiological study was conducted using blood samples from 90 veterinary hospital workers who worked with small animals and from 1000 healthy individuals [17]. Three of the 90 veterinary hospital workers tested positive for anti-SFTSV antibodies in the ELISA, of whom two participants also tested positive for anti-SFTSV neutralizing antibodies. In contrast, none of the healthy individuals tested positive for anti-SFTSV antibodies, suggesting that the prevalence of anti-SFTSV antibodies was higher among veterinary hospital workers than among the general population, and that veterinary hospital workers are at risk of SFTS. However, few studies have assessed the seroprevalence of anti-SFTSV antibodies in veterinary hospital workers [17]. To our knowledge, no previous studies have assessed the risk factors for exposure in high-risk occupational groups through specific epidemiological investigations.

In this study, 64.1% of the participants reported wearing a long-sleeved gown during veterinary treatment, 10.7% reported wearing gloves, and 91.3% reported wearing a mask. In this study, 67% of the participants had experience removing ticks during treatment, and 2.9% of these participants tested positive for anti-SFTSV antibodies with an IFA IgG titer ≥ 1:32. This finding suggests that those working in veterinary hospitals are at an increased risk of tick exposure. Furthermore, 84.5% of the participants had been exposed to animal blood in the last three years. The two participants with neutralizing antibodies and IFA IgG titers ≥ 1:64 reported that they had been exposed to animal blood in the past three years. Moreover, these participants reported that they had not engaged in any risky activity that may have put them in contact with ticks outside of veterinary hospitals in the last three years, suggesting that the veterinary hospital environment increases the likelihood of exposure to animal blood. This observation suggests that the risk of SFTSV exposure may also be high in veterinary hospitals.

Among the participants, all of the veterinarians were aware of SFTS, whereas 29.6% of the animal caregivers were not aware of SFTS. The awareness of possible animal-to-human transmission was significantly lower among the animal caretakers and administrative workers than the veterinarians. This study showed that animal caretakers and administrative staff in veterinary hospitals require training to raise the awareness of SFTS. An epidemiological investigation of the two participants who tested positive for SFTSV-neutralizing antibodies showed that these two participants were unaware of the risk of the blood-borne transmission of SFTS. Protective equipment and contact precautions are crucial when companion animals that may have SFTS are treated, especially in cases of hematemesis or bloody stools.

This study had some limitations. A limited number of veterinary hospitals participated in this study, and only 103 participants were enrolled. Serum was collected mainly at veterinary hospitals in large cities. Including serum samples from veterinary hospital workers in rural districts is likely to provide more meaningful results.

## 5. Conclusions

The seroprevalence of anti-SFTSV antibodies among the 103 veterinary hospital workers was 3.9%, 2.9%, and 1.9% using ELISA, IFA, and PRNT_50_ analyses, respectively. A substantial proportion of the participants including animal caretakers and administrative workers lacked an awareness of SFTS. SFTSV is not transmitted by ticks alone; it can also be transmitted via secondary infection from exposure to the blood of infected animals. Thus, it is important to provide staff working in veterinary hospitals with training concerning standard precautions including the appropriate use of personal protective equipment due to the risk of animal-to-human SFTSV transmission.

## Figures and Tables

**Table 1:** Demographic characteristics and seroprevalence of SFTSV antibodies in the study participants (*n* = 103).

	Number (%)	IFA (Titer ≥ 1:32)	ELISA (OD > 0.92)	PRNT_50_ (Titer ≥ 1:10)
Total	103 (100.0)	3 (2.9)	4 (3.9)	2 (1.9)
Men	21 (20.4)	0 (0.0)	0 (0.0)	0 (0.0)
Women	82 (79.6)	3 (3.7)	4 (4.9)	2 (2.4)
Age (years)				
20–29	46 (44.7)	2 (4.4)	2 (4.4)	1 (2.2)
30–39	39 (37.9)	1 (2.6)	1 (2.6)	1 (2.6)
40–49	15 (14.6)	0 (0.0)	1 (6.7)	0 (0.0)
50–59	3 (2.9)	0 (0.0)	0 (0.0)	0 (0.0)
Residential district				
Gwangju Metropolitan City	18 (17.5)	0 (0.0)	0 (0.0)	0 (0.0)
Seoul Special City	25 (24.3)	0 (0.0)	1 (4.0)	0 (0.0)
Gyeonggi Province	44 (42.7)	3 (6.8)	3 (6.8)	2 (4.5)
Daegu Metropolitan City	11 (10.7)	0 (0.0)	0 (0.0)	0 (0.0)
Jeju Province	5 (4.9)	0 (0.0)	0 (0.0)	0 (0.0)
Occupation				
Veterinarians	42 (40.8)	0 (0.0)	0 (0.0)	0 (0.0)
Animal caretakers (veterinary technicians)	44 (42.7)	2 (4.6)	3 (6.8)	2 (4.6)
Administrative staff	9 (8.7)	1 (11.1)	1 (11.1)	0 (0.0)
Others	8 (7.8)	0 (0.0)	0 (0.0)	0 (0.0)
Animal care career				
<1 year	24 (23.3)	1 (4.2)	0 (0.0)	0 (0.0)
1–3 years	24 (23.3)	1 (4.2)	1 (4.2)	1 (4.2)
3–5 years	19 (18.5)	1 (5.3)	2 (10.5)	1 (5.3)
5–10 years	22 (21.4)	0 (0.0)	0 (0.0)	0 (0.0)
>10 years	14 (13.6)	0 (0.0)	1 (7.1)	0 (0.0)
Average number of contacts with animals per month				
<50	11 (10.9)	0 (0.0)	0 (0.0)	0 (0.0)
50–100	21 (20.8)	0 (0.0)	0 (0.0)	0 (0.0)
100–200	38 (37.6)	3 (7.9)	2 (5.3)	2 (5.3)
200–300	10 (9.9)	0 (0.0)	1 (10.0)	0 (0.0)
>300	21 (20.8)	0 (0.0)	0 (0.0)	0 (0.0)
Isolation rooms in hospital				
No	2 (1.9)	0 (0.0)	0 (0.0)	0 (0.0)
Yes	101 (98.1)	3 (3)	4 (4.0)	2 (2.3)

ELISA, enzyme-linked immunosorbent assay; IFA, immunofluorescence assay; OD, optical density; PRNT_50_, 50% plaque reduction neutralization titer; SFTSV, severe fever with thrombocytopenia syndrome virus.

**Table 2:** Characteristics of the veterinary hospital workers who tested positive for anti-SFTSV antibodies.

Lab ID.	Age	Sex	Study Area	IFA IgG Titer	IFA IgM Titer	ELISA IgG (NP) (OD Value)	PRNT_50_ Titer
2021-126	20	Female	Goyang City, Gyeonggi Province	<1:32	<1:32	0.95	<1:10
2021-164	20	Female	Bundang City, Gyeonggi Province	1:32	<1:32	0.42	<1:10
2021-165	29	Female	Bundang City, Gyeonggi Province	1:256	<1:32	1.23	1:52
2021-167	26	Female	Bundang City, Gyeonggi Province	1:64	<1:32	1.28	1:136
2021-186	20	Female	Seoul Special City	<1:32	<1:32	1.86	<1:10

ELISA, enzyme-linked immunosorbent assay; IFA, immunofluorescence assay; PRNT_50_, 50% plaque reduction neutralization titer; SFTSV, severe fever with thrombocytopenia syndrome virus.

**Table 3:** SFTSV seropositivity according to the awareness of SFTS, gastrointestinal symptoms, and influenza vaccination in 103 veterinary hospital workers.

	Number (%)	IFA (Titer ≥ 1:32)	*p*-Value	ELISA Positive	*p*-Value	PRNT_50_ (Titer ≥ 1:10)	*p*-Value
Awareness of SFTS			0.533		0.199		0.271
Have heard of it but are unfamiliar	40 (38.8)	1 (2.5)		1 (2.5)		0 (0.0)	
Well aware	48 (46.6)	1 (2.1)		1 (2.1)		1 (2.1)	
Never heard of it	15 (14.6)	1 (6.7)		2 (13.3)		1 (6.7)	
Consider that “SFTS can be transmitted from animals to humans”			0.258		0.029		0.513
Yes	72 (69.9)	1 (1.4)		1 (1.4)		1 (1.4)	
No	3 (2.9)	0 (0.0)		1 (33.3)		0 (0.0)	
Unsure	28 (27.2)	2 (7.1)		2 (7.1)		1 (3.6)	
Consider that “SFTS is a zoonotic infectious disease”			0.304		0.198		0.566
Yes	68 (66)	1 (1.5)		1 (1.5)		1 (1.5)	
No	3 (2.9)	0 (0.0)		0 (0.0)		0 (0.0)	
Unsure	32 (31.1)	2 (6.3)		3 (9.4)		1 (3.1)	
Gastrointestinal symptoms and fever in past three years			0.145		0.247		0.428
No	78 (75.7)	1 (1.3)		2 (2.6)		1 (1.3)	
Yes	25 (24.3)	2 (8.0)		2 (8.0)		1 (4.0)	
Flu vaccination in 2019			0.279		0.122		0.579
Yes	16 (15.5)	1 (6.3)		1 (6.3)		0 (0.0)	
No	67 (65.1)	1 (1.5)		1 (1.5)		1 (1.5)	
Unsure	20 (19.4)	1 (5)		2 (10.0)		1 (5.0)	
Flu vaccination in 2020			0.603		0.034		0.458
Yes	5 (4.9)	0 (0.0)		1 (20.0)		0 (0.0)	
No	76 (73.8)	2 (2.6)		1 (1.3)		1 (1.3)	
Unsure	22 (21.4)	1 (4.6)		2 (9.1)		1 (4.6)	

ELISA, enzyme-linked immunosorbent assay; IFA, immunofluorescence assay; PRNT_50_, 50% plaque reduction neutralization titer; SFTS, severe fever with thrombocytopenia syndrome; SFTSV, severe fever with thrombocytopenia syndrome virus.

**Table 4:** SFTS awareness according to occupation.

		Veterinarians, Number (%)	Animal Caretakers (Veterinary Technicians), Number (%)	Administrative Staff, Number (%)	Others, Number (%)
SFTS awareness	Well aware	35 (83.3)	11 (25.0)	1 (11.1)	1 (12.5)
	Have heard of it but are unfamiliar	7 (16.7)	20 (45.5)	7 (77.8)	6 (75.0)
	Have never heard of it	0 (0.0)	13 (29.6)	1 (11.1)	1 (12.5)
	*p*-value *	<0.001			
Consider that “SFTS can be transmitted from animals to humans”	Yes	41 (97.6)	23 (52.3)	4 (44.4)	4 (50.0)
	No	0 (0.0)	1 (2.3)	1 (11.1)	1 (12.5)
	Unsure	1 (2.4)	20 (45.5)	4 (44.4)	3 (37.5)
	*p*-value *	<0.001			

* Fisher’s exact test. SFTS, severe fever with thrombocytopenia syndrome.

**Table 5:** SFTS seroprevalence according to preventive behaviors during treatment and exposure history.

	Number (%)	IFA (Titer ≥ 1:32)	*p*-Value	ELISA (OD > 0.92)	*p*- Value	PRNT_50_ (Titer ≥ 1:10)	*p*-Value
Wore long-sleeved gown during treatment			>0.999		0.131		>0.999
No	37 (35.9)	1 (2.7)		3 (8.1)		1 (2.7)	
Yes	66 (64.1)	2 (3.0)		1 (1.5)		1 (1.5)	
Wore gloves during treatment			>0.999		>0.999		>0.999
No	92 (89.3)	3 (3.3)		4 (4.4)		2 (2.2)	
Yes	11 (10.7)	0 (0.0)		0 (0.0)		0 (0.0	
Wore a mask during treatment			>0.999		0.310		>0.999
No	9 (8.7)	0 (0.0)		1 (11.1)		0 (0.0)	
Yes	94 (91.3)	3 (3.2)		3 (3.2)		2 (2.1)	
Wore goggles during treatment			>0.999		>0.999		>0.999
No	102 (99)	3 (2.9)		4 (3.9)		2 (2.0)	
Yes	1 (1)	0 (0.0)		0 (0.0)		0 (0.0)	
Showered or bathed every day after work			0.535		0.215		0.398
No	23 (22.3)	1 (4.4)		2 (8.7)		1 (4.4)	
Yes	80 (77.7)	2 (2.5)		2 (2.5)		1 (1.3)	
Wore protective clothing (for example, a disposable apron) for individual treatment			>0.999		>0.999		>0.999
No	94 (91.3)	3 (3.2)		4 (4.3)		2 (2.1)	
Yes	9 (8.7)	0 (0.0)		0 (0.0)		0 (0.0)	
Hand hygiene for individual treatment			>0.999		0.473		>0.999
No	15 (14.6)	0 (0.0)		1 (6.7)		0 (0.0)	
Yes	88 (85.4)	3 (3.4)		3 (3.4)		2 (2.3)	
Had experience with removing ticks			0.704				0.553
No	13 (12.6)	0 (0.0)		1 (7.7)	0.145	0 (0.0)	
Yes	69 (67)	2 (2.9)		1 (1.5)		1 (1.5)	
Not applicable	21 (20.4)	1 (4.8)		2 (9.5)		1 (4.8)	
Wore gloves when removing ticks			>0.999		0.139		>0.999
No	24 (23.3)	1 (4.2)		0 (0.0)		0 (0.0)	
Yes	49 (47.6)	1 (2.0)		1 (2.0)		1 (2.0)	
Not applicable	30 (29.1)	1 (3.3)		3 (10.0)		1 (3.3)	
Used tools (forceps) when removing ticks			0.682		0.733		>0.999
No	11 (10.7)	0 (0.0)		0 (0.0)		0 (0.0)	
Yes	65 (63.1)	3 (4.6)		2 (3.1)		2 (3.1)	
Not applicable	27 (26.2)	0 (0.0)		2 (7.4)		0 (0.0)	
Exposure to animal blood in the last three years			0.401		0.497		>0.999
No	16 (15.5)	1 (6.3)		1 (6.3)		0 (0.0)	
Yes	87 (84.5)	2 (2.3)		3 (3.5)		2 (2.3)	
Cases of SFTS infection in animals treated			0.414		0.317		0.456
Unsure	48 (46.6)	2 (4.2)		3 (6.3)		1 (2.1)	
No	36 (35)	0 (0.0)		0 (0.0)		0 (0.0)	
Yes	19 (18.5)	1 (5.3)		1 (5.3)		1 (5.3)	
Found tick when in contact with animals			>0.999		0.124		
Unsure	6 (5.8)	0 (0.0)		1 (16.7)		0 (0.0)	
No	18 (17.5)	0 (0.0)		1 (5.6)		0 (0.0)	
Yes	79 (76.7)	3 (3.8)		2 (2.5)		2 (2.5)	
Ever been bitten by a tick			>0.999		1.000		
No	100 (97.1)	3 (3.0)		4 (4.0)		2 (2.0)	
Yes	3 (2.9)	0 (0.0)		0 (0.0)		0 (0.0)	

## Data Availability

All relevant data are within the manuscript and the files in the Supplementary Materials.

## Supplementary Materials

The following supporting materials can be downloaded at https://www.mdpi.com/article/10.3390/v15030609/s1, Supplementary Figure S1: Results of the PRNT in serum samples from 2 participants who were PRNT positive; Supplementary Figure S2: Hematemesis of the Samoyed dog suspected of being infected with SFTS who came into contact with the 2 participants who tested positive for SFTSV-neutralizing antibodies.
