# Supplementary material for: The Seroprevalence of Severe Fever with Thrombocytopenia Syndrome: An Epidemiological Study of Korean Veterinary Hospital Workers
Source: Viruses. 2023 Feb 23;15(3):609. doi: 10.3390/v15030609 (PMC10052674; doi:10.3390/v15030609)
Supplement: Supplementary file 1 [file viruses-15-00609-s001.zip › viruses-2023679-supplementary.pdf]

Supplementary Data

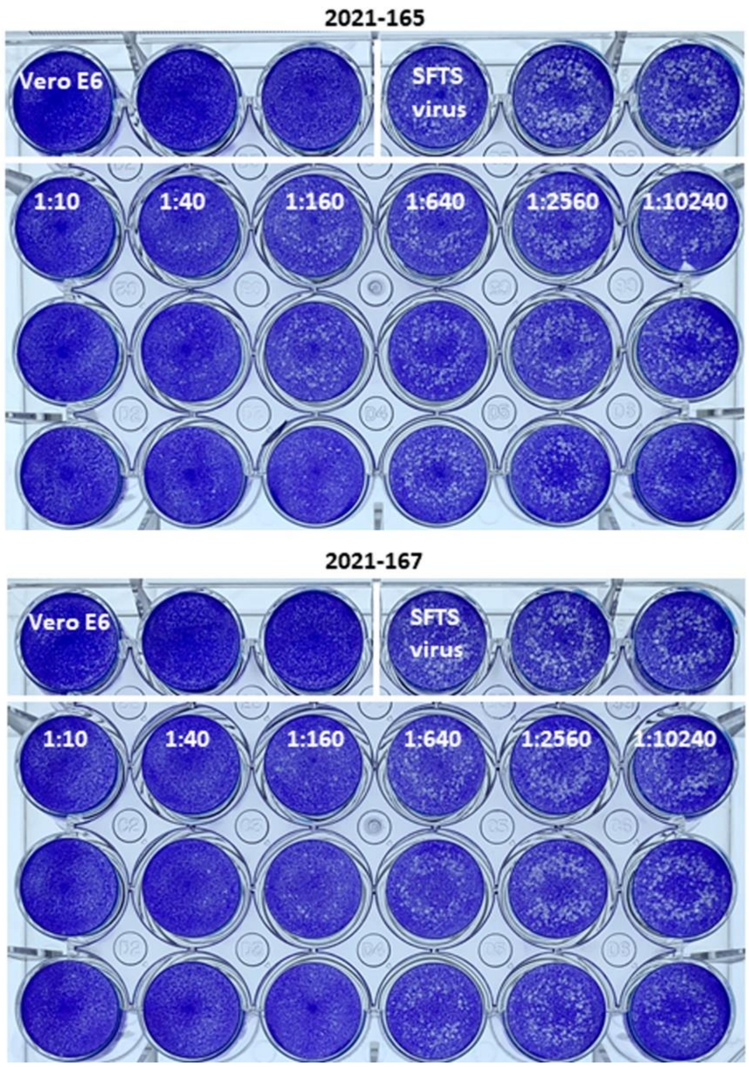

Supplementary Figure S1. Results of the PRNT in serum samples from 2 participants who were PRNT positive.

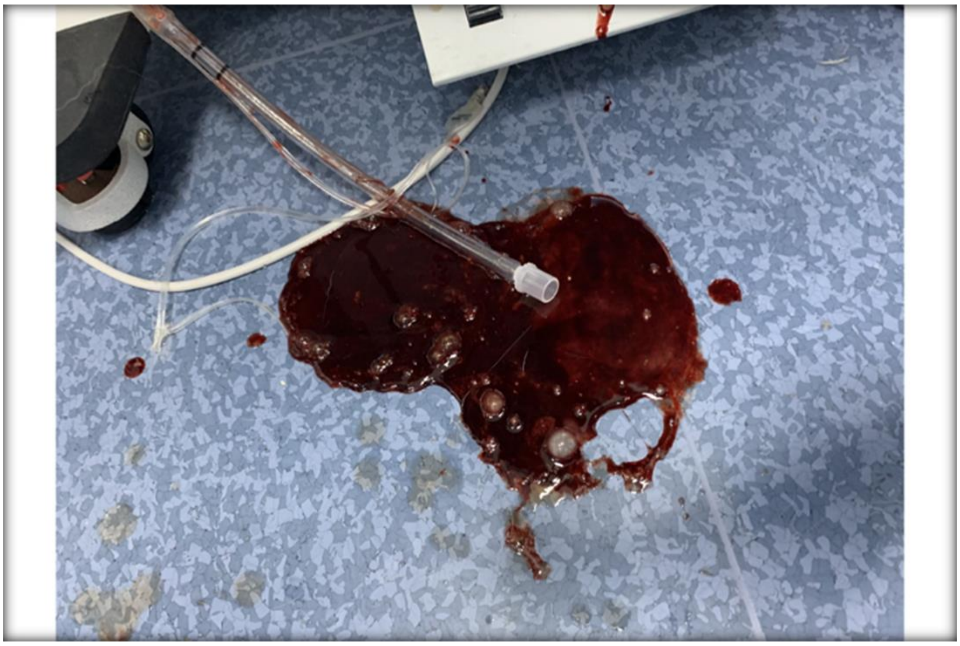

Supplementary Figure S2. Hematemesis of the Samoyed dog suspected of being infected with SFTS who came into contact with the 2 participants who tested positive for SFTSV-neutralizing antibodies.
